# Detrimental Impact of Energy Drink Compounds on Developing Oligodendrocytes and Neurons

**DOI:** 10.3390/cells8111381

**Published:** 2019-11-03

**Authors:** Meray Serdar, Annika Mordelt, Katharina Müser, Karina Kempe, Ursula Felderhoff-Müser, Josephine Herz, Ivo Bendix

**Affiliations:** Department of Paediatrics I, Neonatology & Experimental perinatal Neurosciences, University Hospital Essen, University Duisburg-Essen, 45147 Essen, Germany

**Keywords:** energy drinks, caffeine, taurine, neuron, oligodendrocytes

## Abstract

The consumption of energy drinks is continuously rising, particularly in children and adolescents. While risks for adverse health effects, like arrhythmia, have been described, effects on neural cells remain elusive. Considering that neurodevelopmental processes like myelination and neuronal network formation peak in childhood and adolescence we hypothesized that developing oligodendrocytes and neurons are particularly vulnerable to main energy drink components. Immature oligodendrocytes and hippocampal neurons were isolated from P0-P1 Wistar rats and were incubated with 0.3 mg/mL caffeine and 4 mg/mL taurine alone or in combination for 24 h. Analysis was performed immediately after treatment or after additional three days under differentiating conditions for oligodendrocytes and standard culture for neurons. Oligodendrocyte degeneration, proliferation, and differentiation were assessed via immunocytochemistry and immunoblotting. Neuronal integrity was investigated following immunocytochemistry by analysis of dendrite outgrowth and axonal morphology. Caffeine and taurine induced an increased degeneration and inhibited proliferation of immature oligodendrocytes accompanied by a decreased differentiation capacity. Moreover, dendritic branching and axonal integrity of hippocampal neurons were negatively affected by caffeine and taurine treatment. The negative impact of caffeine and taurine on developing oligodendrocytes and disturbed neuronal morphology indicates a high risk for disturbed neurodevelopment in children and adolescents by excessive energy drink consumption.

## 1. Introduction

The consumption of energy drinks has exponentially increased due to the expectation of increased mental and physical performance. This makes them particularly attractive for children, adolescents, and young adults at reproductive age, who are the main consumers of energy drinks, comprising 30 to 50% of all consumers [1,2]. A significant increase in adolescents who died after excessive consumption of energy drinks in combination with or without alcohol was noted in recent years [3]. Furthermore, several studies reported negative health effects of short- and long-term consumption of energy drinks, such as aggressive behaviour, arrhythmia, increased heart rate, and sleep disturbances [4,5,6]. These effects were mainly attributed to the high caffeine and sugar concentrations [3,7]. To date there are no studies analysing a potential risk of energy drink consumption on key neurodevelopmental processes of the central nervous system (CNS). Childhood and adolescence is a period of rapid body growth but also an important period of brain development. White matter volume, i.e., myelination increases linearly with increasing age, beginning at the end of the second trimester and continuing into the third decade of life [8,9,10]. Furthermore, grey matter volume and formation, reorganization of neuronal projections reach its maximum during childhood and adolescence [9].

Considering the continuously rising number of children and adolescents consuming energy drinks and the fact that brain development is in a crucial phase, neuronal and glial cell types of the still immature brain might be particularly vulnerable to high doses of energy drink compounds. Al Basher et al. and Reis et al. provided first indications for detrimental effects of energy drinks on the developing brain resulting in anxiety disorders in later life [4,11]. With regard to the molecular components of energy drinks, potentially harmful to neural cells, it was suggested that caffeine and taurine either alone or in combination might be involved [12]. However, these data were obtained in the human neuroblastoma cell line SH-SY5Y. Effects on primary developing CNS cell types remain unclear.

In the present study, we investigated the impact of caffeine and taurine alone or in combination on primary immature oligodendrocytes and hippocampal neurons, focusing on degeneration, proliferation, and differentiation capacity of immature oligodendrocytes as well as on dendritic branching and axonal morphology of hippocampal neurons.

## 2. Materials and Methods

### 2.1. Drugs

Caffeine and taurine were purchased from Sigma Aldrich (Taufkirchen, Germany). Both components were dissolved to a final concentration of 0.3 mg/mL caffeine and 4 mg/mL taurine in the appropriate medium of oligodendrocytes and neurons. The selected concentrations are based on a previous report in neuronal SH-SY5Y cells [12].

### 2.2. Primary Cell Culture

#### 2.2.1. Oligodendrocytes

Neonatal rat mixed glia cells were isolated from P0–P1 old Wistar rats as previously described [13,14,15,16]. Cells were cultured in Dulbecco’s modified Eagle’s medium (DMEM, Gibco, Erlangen, Germany) supplemented with 20% foetal calf serum, 1% penicillin/streptomycin. After 10–12 days, immature oligodendrocytes, attached to the astrocyte layer of the mixed glia culture, were isolated by shaking overnight at 230 rpm. The collected cells were pre-plated for 30 min at 5% CO_2_ and 37 °C to remove contaminating microglia and astrocytes. Floating cells were collected in proliferation medium (DMEM, 1×B27 (Gibco), 10 ng/mL plated derived growth factor (PDGF, PanBiotech, Aidenbach, Germany), 10 ng/mL basic fibroblast growth factor (bFGF, PanBiotech)) and plated on poly-dl-ornithine-coated (Sigma-Aldrich, Taufkirchen, Germany) cover slips or culture plates (100,000 cells/mL). At this stage the purity of culture was approximately around 95%, assessed by expression of the immature oligodendrocyte surface progenitor marker A2B5 [17]. Oligodendrocyte precursor cells were cultivated in proliferation medium for 3 days before treatment with 0.3 mg/mL caffeine, 4 mg/mL taurine, and the combination of both for 24 h at 5% CO_2_ and 37 °C in humidified air. To quantify the effect of caffeine and taurine on myelination capacity of oligodendrocytes, cells were cultivated in differentiation medium (DMEM, 1× B27, 10 ng/mL ciliary neurotrophic factor (CNTF, PanBiotech), 15 µM triiodothyronine (T3, Sigma-Aldrich)) for another 3 days after treatment. Cells were lysed for western blot analysis or fixed with 2% paraformaldehyde (PFA) for analysis via immunocytochemistry (ICC).

#### 2.2.2. Hippocampal Neurons

In the present study, neurons were isolated from the hippocampus, the main structure of learning and memory formation involving adult neurogenesis and synaptogenesis [18], which has been suggested to be impaired by energy drink components [19]. Hippocampal neurons were isolated from P0–P1 old Wistar rats according to described methods [20]. Briefly, hippocampi were carefully dissected from the cerebrum and enzymatically digested with trypsin (2.5%, Sigma-Aldrich) and deoxyribonuclease (DNase, 1%, Sigma-Aldrich). Subsequently the hippocampi were mechanically disrupted by a glass pipette followed by pre-plating of the cell suspension for 30 min to remove contaminating astrocytes. 80,000 cells/mL were seeded on double coated poly-l-lysine and laminin (Sigma-Aldrich) Falcondishes (Amsterdam, Netherlands) in neurobasal media (NB, Gibco) containing B27, 1% glutamine, and 1% penicillin/streptomycin for 24 h. Afterwards, neuron-enriched cultures were treated with 3 µM cytosine beta-d-arabinofuranosyl cytosine (AraC, Sigma-Aldrich) for 48 h to inhibit proliferation of contaminating astrocytes [20], resulting in a purity of approximately 80%. Neurons were treated as described for oligodendrocyte cultures. Appropriate controls were cultivated under standard culture conditions in NB medium. Both substances were dissolved in the NB medium. Cells were fixated with 2% PFA immediately after the treatment or after cultivation for another 3 days in NB medium.

### 2.3. Western-Blot Analysis

For immunoblot analysis oligodendrocytes were plated in 6-well plates. Cells of three wells for each condition were lysed in ice-cooled radioimmunoprecipitation assay (RIPA) buffer containing protease inhibitors. The homogenate was centrifuged at 14,000× *g* (4 °C) for 10 min. The protein concentration in the supernatant cytosolic extract was determined using the bicinchoninic acid assay (BCA assay; Thermo Fisher Scientific, Erlangen, Germany). Ten µg of protein were denaturated in Laemmli sample buffer at 95 °C for 5 min. Proteins were separated by 15% sodium dodecyl sulphate polyacrylamide gel electrophoresis, and blotted onto nitrocellulose membranes (0.2 µm pore, Sigma-Aldrich). Equal loading and transfer of proteins was confirmed by staining with Ponceau S solution (Sigma-Aldrich). Five percent nonfat dry milk was used for blocking of nonspecific antibody binding in Tris buffered saline/0.1% Tween (TBST) at room temperature for 60 min. Membranes were incubated overnight (4 °C) with the primary monoclonal rabbit anti-cleaved Caspase-3 (cCaspase-3) antibody (1:1000, Cell Signaling Technology, Frankfurt am Main, Germany; molecular weight 19 kDa) or mouse anti-glyceraldehyde 3-phosphate dehydrogenase (GAPDH) antibody (1:50,000; Sigma-Aldrich; molecular weight 37 kDa) in 5% nonfat dry milk in TBST. Horseradish peroxidase-conjugated secondary antibodies (DAKO, Hamburg, Germany) were diluted 1:5000 (anti-mouse) or 1:2000 (anti-rabbit) in 5% non-fat dry milk in TBST. For visualization and densitometric analysis ChemiDoc XRS^+^ imaging system and ImageLab software (6.0.1, Bio-Rad, Munich, Germany) was used. Density ratios between cCaspase-3 protein and the reference protein GAPDH were calculated for each (=1) sample per experiment. These ratios were normalized to control (i.e., without treatment) per experiment. The mean of four independent experiments was used for graphical presentation. 

### 2.4. Immunocytochemistry

Following fixation, cells were incubated with blocking solution (5% normal goat serum in 0.1% Triton X-100 in phosphate-buffered-solution (PBS)) for 1 h at room temperature followed by incubation with primary antibodies in PBS with 5% goat serum at 4°C overnight. The following antibodies were used: anti-oligodendrocyte transcription factor 2 (polyclonal rabbit anti-Olig2, 1:500; monoclonal mouse anti-Olig2, 1:300, Millipore, Darmstadt, Germany), anti-A2B5 (monoclonal mouse anti-A2B5, 1:500, Millipore, Germany), anti-proliferating cell nuclear antigen (polyclonal rabbit anti-PCNA, 1:800, Cell Signaling Technology), anti-myelin basic protein (monoclonal mouse anti-MBP, 1:500, Covance, Munich, Germany), anti-microtubuli associated protein 2 (polyclonal mouse anti-Map2, 1:500, Sigma-Aldrich), and anti-TAU (polyclonal rabbit anti-TAU, 1:500, GeneTex, Germany). Specific antibody binding was visualized by incubation with the appropriate secondary antibodies (anti-mouse Alexa Fluor 555; anti-mouse Alexa Fluor 488; anti-rabbit Alexa Fluor 555; anti-rabbit Alexa Fluor 488; anti-rabbit Alexa Fluor 647, Invitrogen, Erlangen, Germany; all 1:1000) for 1 h at room temperature. Nuclei were counterstained with 4′,6-diamidino-2-phenylindole (DAPI, 1 µg/mL). Cover slips were mounted onto glass slides with DAKO Fluorescent Mounting Medium and kept in the dark at 4 °C. Cells were analysed via confocal microscopy (A1plus, Eclipse Ti, with NIS Elements AR software, Nikon, Düsseldorf, Germany). 

### 2.5. Confocal Microscopy and Quantitative Analysis

Analysis was performed with confocal microscopy (A1plus, Eclipse Ti, with NIS Elements AR software, Nikon) using a 10× objective. Four laser lines (laser diode, 405 nm; Ar laser, 514 nm; G-HeNe-laser, 543 nm and Rh-laser 647nm) and three different filters (450/50-405 LP, 515/20-540 LP, 585/65-640 LP) were used for image acquisition. Oligodendrocytes were analysed in a total of 15 random fields of view (each 1.6 mm^2^) derived from three independent experiments (5 images per experiment and group). The number of total (Olig2) and proliferating (PCNA) oligodendrocytes as well as the area of A2B5 were analysed automatically using the NIS Elements AR software 4.0 (Nikon). Since reductions of total MBP-positive cells after initiation of differentiation might have resulted from a total reduction of immature A2B5-positive oligodendrocytes after acute treatment, i.e., before initiation of differentiation, the percentage of MBP-positive cells of total Olig2-positive cells was quantified. As, under differentiating conditions, immature oligodendrocytes do not proliferate and substances were not present during this culture period, percentage values of MBP+ cells provide information about the general capacity of all initially surviving oligodendrocytes independent of acute treatment effects on cell numbers. To investigate morphological changes of neurons, 8–10 random fields (each 0.395 mm^2^) per experiment were analysed in Map2/TAU co-staining by using the 20× objective. The images were converted to Tiff-images using the NIS Elements AR software 4.0 (Nikon, Germany). Analysis of dendrite branches and length was performed with ImageJ (NIH, Java 1.8.0) using the NeuronJ plugin [21].

### 2.6. Statistical Analysis

Data are expressed as scatter plots with bars including mean values with standard deviation (SD). Data were analysed using GraphPad Prism 6 (Statcon, Witzenhausen, Germany). Differences between groups were determined by one-way analysis of variance (one-way ANOVA) followed by Bonferroni post hoc test for multiple comparison. *p*-values < 0.05 were considered as statistically significant. Detailed results of statistical analysis are provided in Supplementary Tables S1 and S2 in the Supplementary Material.

## 3. Results

### 3.1. The Main Compounds of Energy Drinks Caffeine and Taurine Induce Cell Degeneration and Inhibit Proliferation of Immature Oligodendrocytes

**Figure 1:**
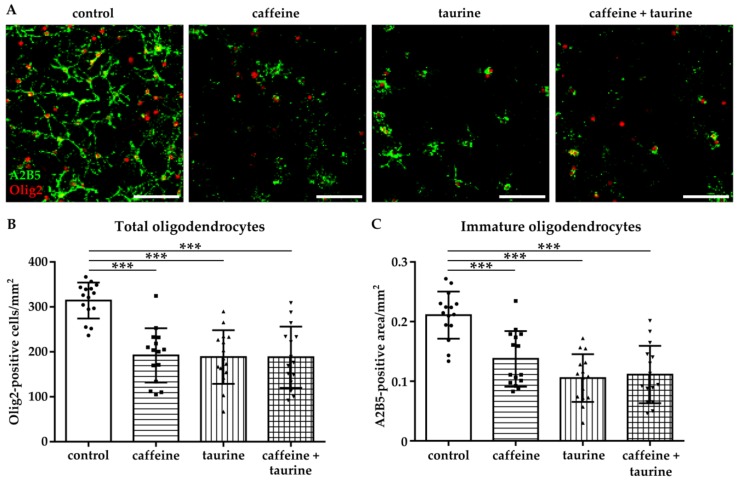
Caffeine and taurine alone or in combination induce immature oligodendrocyte cell loss. After three days under standard culture conditions primary immature oligodendrocytes were incubated with or without 0.3 mg/mL caffeine, 4 mg/mL taurine, and the combination of both for 24 h. (**A**) Representative images of immunofluorescence staining of immature oligodendrocytes (A2B5 green, Olig2 red). (**B**) Quantification of Olig2 positive cells. The density of immature oligodendrocytes was determined by measuring the A2B5 positive area (**C**). Data are derived from 15 images per group out of three independent experiments (depicted by circle = control, square = caffeine, triangle up = taurine, triangle down = caffeine + taurine). Scale bar 400 µm. *** *p* < 0.001, one-way ANOVA followed by Bonferroni post hoc test.

The selected treatment design with 0.3 mg/mL caffeine and 4 mg/mL taurine for 24 h is mainly based on a previous in vitro study on neural cells [12] and concentrations in commercially available energy drinks considering the increasing rise of high acute and high chronic consumers [22]. Immediately after treatment, oligodendrocytes were stained for the pan-oligodendrocyte marker Olig2 and the immature cell marker A2B5 (Figure 1A). We observed a significant reduction of immature oligodendrocytes by 40–50% in the presence of caffeine, taurine, and the combination of both (Olig2/mm^2^: F_3, 56_ = 17.33; mean: control 314.2, caffeine 192.2, taurine 188.6, caffeine + taurine 188.1; A2B5 area/mm^2^: F_3, 56_ = 18.43; mean: control 0.21, caffeine 0.14, taurine 0.11 and caffeine +taurine 0.11, Figure 1B,C).

**Figure 2:**
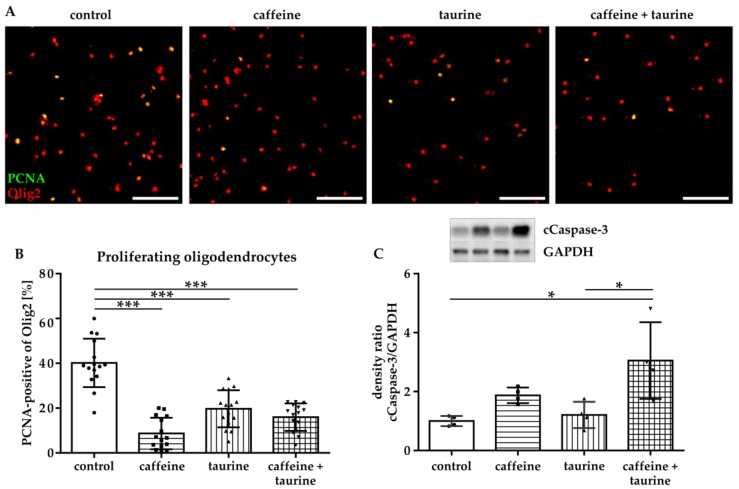
Immature oligodendrocyte cell loss is caused by a decreased proliferation and increased degeneration induced by caffeine and taurine. After treatment with or without 0.3 mg/mL caffeine and 4 mg/mL taurine alone or in combination, cells were analysed for proliferation via immunocytochemistry and for apoptosis via western blot. (**A**) Representative images of immunofluorescence staining of proliferating oligodendrocytes (PCNA green, Olig2 red). The number of proliferating oligodendrocytes was quantified in 15 images per group derived from three independent experiments (**B**) Scale bar 400 µm. Ratio of cCaspase-3 and GAPDH protein expression was analyzed in protein lysates of cultured oligodendrocytes derived from four independent experiments (**C**), depicted by circle = control, square = caffeine, triangle up = taurine, triangle down = caffeine + taurine). *p < 0.05, *** *p* < 0.001, one-way ANOVA followed by a Bonferroni post hoc test.

Since reduced cell density may have been caused either by increased cell degeneration and/or reduced proliferation, we analysed proliferation of immature oligodendrocytes via immunocytochemistry for the marker PCNA (Figure 2A,B). These analyses demonstrated a significantly reduced proliferation of immature oligodendrocytes incubated with caffeine, taurine, and the combination of both (% PCNA-positive Olig2: F_3, 56_ = 40.10, mean: control 40.15, caffeine 8.67, taurine 19.71 and caffeine+ taurine 15.97, Figure 2B). Analysis of apoptosis by western blot for cleaved-Caspase-3 [23] revealed a significant three-fold increase in the combined treatment compared to control (F_3, 12_ = 6.886; mean cCaspase-3/GAPDH ratio: caffeine + taurine 3.05 compared to control (=1); Figure 2C). Of note, single taurine treatment did not affect apoptotic cell death, resulting in a significant difference between taurine single and the combined treatment with caffeine (Figure 2C).

### 3.2. Differentiation Capacity of Oligodendrocytes is Reduced by Caffeine and Taurine

**Figure 3:**
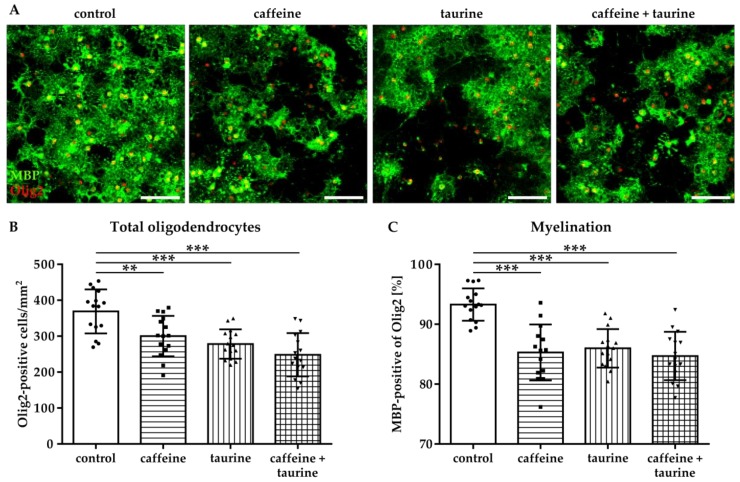
Myelination capacity of immature oligodendrocytes is decreased by caffeine and taurine. Primary immature oligodendrocytes were treated with or without 0.3 mg/mL caffeine, 4 mg/mL taurine, and the combination of both for 24 h followed by media exchange to differentiating conditions for another three days. (**A**) Representative images of immunofluorescence staining for the pan-oligodendrocyte marker Olig2 (red) and the myelination marker MBP (green) to visualize mature oligodendrocytes. The density of oligodendrocytes was quantified by counting Olig2 positive cells (**B**). MBP-positive oligodendrocytes were quantified by subtracting MBP-negative cells from the total Olig2-positive cell number. (**C**). Data are derived from 15 images per group out of three independent experiments, depicted by circle = control, square = caffeine, triangle up = taurine, triangle down = caffeine + taurine. Scale bar 400 µm. ** *p* < 0.01, *** *p* < 0.001, one-way ANOVA followed by a Bonferroni post hoc test.

To investigate whether the treatment of the main energy drink ingredients caffeine and taurine influence differentiation capacity of oligodendrocytes, we analysed the expression of MBP three days after substance treatment and induction of differentiation (Figure 3A). In accordance with acute treatment effects the number of oligodendrocytes remained reduced under differentiating, i.e., non-proliferating conditions (Figure 3B). In addition to an acute decrease in immature A2B5-positive oligodendrocytes (Figure 1), differentiation capacity of surviving oligodendrocytes was significantly reduced to 85% by caffeine and taurine with the single as well as combined treatment (F_3, 56_ = 17.43; mean % MBP of total Olig2: control 93.29, caffeine 85.29, taurine 85.98 and caffeine + taurine 84.69; Figure 3C).

### 3.3. Caffeine and Taurine Reduce Dendrite Branching Immediately after Treatment

**Figure 4:**
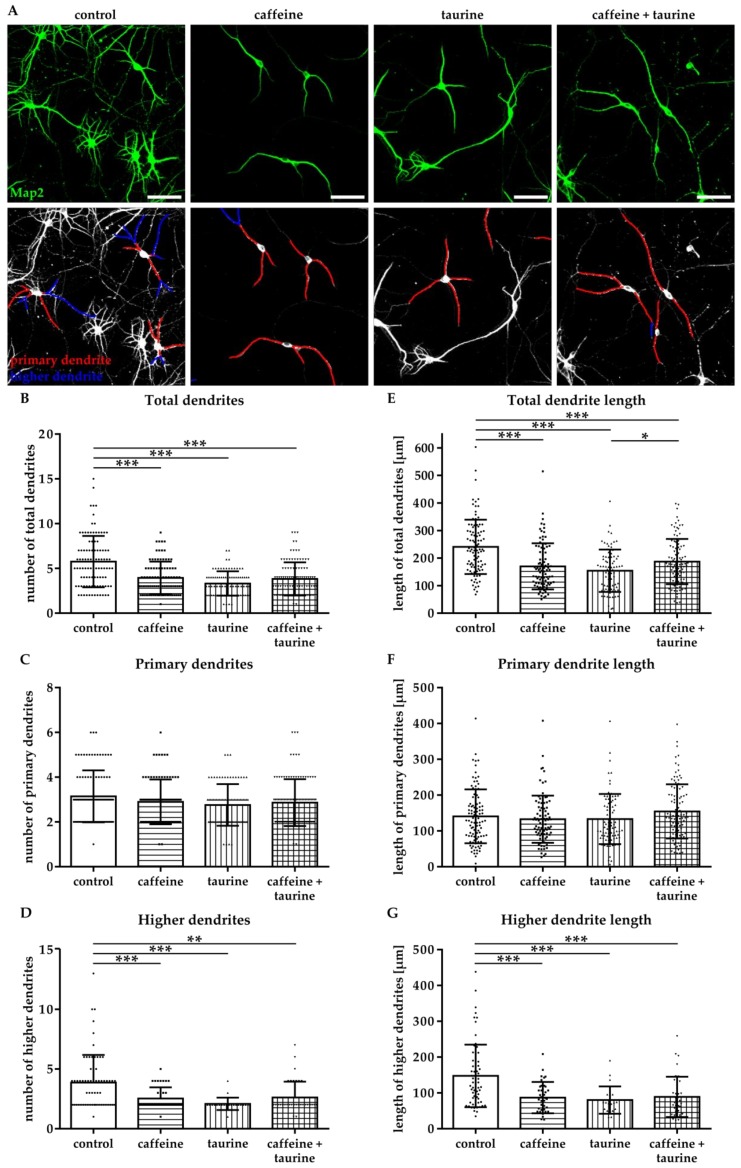
Caffeine and taurine alone or in combination reduce branching of dendrites in primary hippocampal neurons. Following three days in culture primary hippocampal neurons were incubated with or without 0.3 mg/mL caffeine, 4 mg/mL taurine and the combination of both for 24 h. (**A**) Dendrites were visualized by immunofluorescence staining for microtubule-associated protein 2 (Map2, green). Morphological changes were measured by defining primary dendrites (**A**, bottom row, red) and higher dendrites (**A**, bottom row, blue). The amount of total (**B**), primary (**C**), and higher (**D**) dendrites was quantified; subfigures (**E**–**G**) represent results of quantification of dendritic lengths, respectively. Data are derived from 25–30 images per group out of three independent experiments (n = 83–103 cells per group, depicted by circle = control, square = caffeine, triangle up = taurine, triangle down = caffeine + taurine). Scale bar 100 µm. * *p* < 0.05, ** *p* < 0.01, *** *p* < 0.001, one-way ANOVA followed by a Bonferroni post hoc test.

The effect of energy drink components on neuronal network formation was analysed in primary hippocampal neurons. Immediately after treatment with caffeine and taurine alone or in combination, the number of Map2-positive neurons was significantly reduced (Supplementary Figure S1). To get further insight into potential effects on neuronal network formation, neuronal dendrites were analysed (Figure 4A). Two types of dendrites were analysed, primary dendrites attached to the soma and higher dendrites, branching directly from primary dendrites using the NeuronJ plugin [21] of ImageJ software (Figure 4A). We detected a significantly reduced number of total dendrites in all treatment groups compared to controls (F_3, 370_ = 25.07; mean: control 5.76, caffeine 3.94, taurine 3.31 and caffeine + taurine 3.83, Figure 4B). The reduction of total dendrites was mainly caused by a reduction of higher dendrites, demonstrated by a significant reduction by 20–40% in treated cells compared to controls (F_3, 160_ = 9.70; mean: control 3.87, caffeine 2.51, taurine 2.09 and caffeine + taurine 2.62; Figure 4D). In addition to the number of dendrites their length was similarly reduced (Figure 4E). Again, higher dendrites were primarily affected, as shown by significantly reduced lengths to 55–60% compared to controls, while no significant differences were observed for primary dendrites (Figure 4F,G).

### 3.4. Prolonged Disturbance of Dendrite and Axonal Morphology Following Short-Term Caffeine and Taurine Treatment

**Figure 5:**
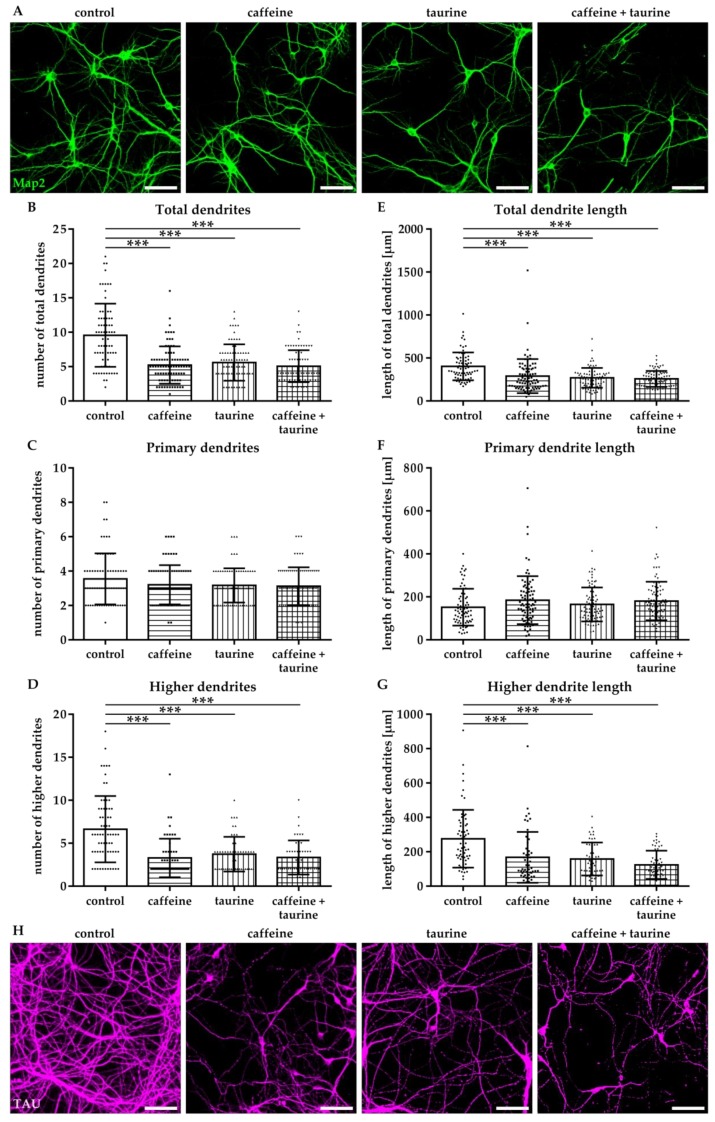
Caffeine and taurine lead to sustained reduced dendritic branching and disturbed axonal network formation. After three days in culture primary hippocampal neurons were treated with or without 0.3 mg/mL caffeine, 4 mg/mL taurine and the combination of both for 24 h followed by cultivation under standard culture conditions for another three days. (**A**) Representative images of Map2 (green) stained dendrites. Quantifications of the amount of total, primary and higher dendrites, as well as their lengths are shown in subfigures (**B**–**G**). Data are derived from 25–30 images per group out of three independent experiments (n = 75–82 cells per group, depicted by circle = control, square = caffeine, triangle up = taurine, triangle down = caffeine + taurine). Morphological changes of axons were visualized by immunofluorescence staining for TAU (**H**). Scale bar 100 µm. *** *p* < 0.001, one-way ANOVA followed by a Bonferroni post hoc test.

To investigate whether the acute effect of caffeine and taurine on dendrite morphology correlates with changes in neuronal development and network formation, neurons were cultivated under standard culture conditions for another three days following substance treatment. According to results after immediate analysis at the acute time point, the number of Map2-positive higher dendrites and their length were significantly reduced to an average of 50% compared to controls (F_3, 211_ = 21.75; mean: control 6.73, caffeine 3.35, taurine 3.73 and caffeine + taurine 3.41; Figure 5A–G). To verify whether disturbed dendrite branching was associated with changes in axonal morphology we analysed TAU-expression by immunocytochemistry. While we observed a densely packed network of intact axons without or occasionally appearing breaks in the control group, caffeine and taurine treated neurons demonstrated more axon breaks and pearl-like structures (Figure 5H), previously described as accumulations of microtubule associated and molecular motor proteins as well as organelles and vesicles, as an indicator for defective and damaged neurons [12,24]. 

## 4. Discussion

The widespread consumption of energy drinks became popular among adolescents below the age of 25 in recent years. Taking the fact that brain development in childhood/adolescence is not completed [9], neuronal and glial cell types might be particularly vulnerable to high doses of energy drink ingredients. The goal of the present study was to investigate the effects of the main energy drink components caffeine and taurine on neuronal cells and immature oligodendrocytes. Using purified primary cell cultures, we demonstrated that the energy drink ingredients caffeine and taurine induce degeneration and reduce proliferation of immature oligodendrocytes, accompanied by a decreased myelination capacity. Furthermore, caffeine and taurine impaired neuronal network formation revealed by reduced dendrite branching and fragmented axons. The present study suggests detrimental effects of increased energy drink consumption particularly during brain development which lasts into early adulthood.

The observed adverse health effects of excessive energy drink consumption have been mainly attributed to high caffeine concentrations. We demonstrated that caffeine inhibits proliferation and myelination capacity of primary oligodendrocytes. These results seem to contradict previous findings in models of adult neurodegenerative diseases like Parkinson and Alzheimer’s disease [25]. Furthermore, in vitro studies on hypoxia-induced degeneration of oligodendrocytes demonstrated protective effects by caffeine treatment [26]. In the context of brain injury to the developing brain, Endesfelder et al. also described neuroprotective effects of caffeine revealed by reduced oxidative stress, decreased neuronal degeneration, and diminished inflammation after oxygen-induced toxicity in vivo [27,28]. However, caffeine concentrations, used in these previous studies, have been much lower than the ones used in the present in vitro study, i.e., Endesfelder et al. used a 30-fold lower concentration [28].

Besides differences in dose, long duration of caffeine exposure might explain our findings. This is supported by recent studies in mouse epidermal cells and human neuroblastoma cells that suggested that even low caffeine concentrations for 24 h may induce apoptotic cell degeneration through caspase 3-induced signalling-pathways [29,30]. In the present study we decided for 24 h exposure according to the previous studies by Zeidán-Chuliá et al. and Doyle et al. [12,31]. Furthermore, caffeine is absorbed very fast, reaching peak plasma concentrations after 30–120 min [32]. Based on its hydrophobic structure it can easily pass the blood–brain barrier [33]; and CSF levels are described to be similar to plasma concentrations [34]. The different half-life times of caffeine with 100 h in preterms, compared to 3–5 h in adults [34], suggest an exponential decrease of the caffeine half-life time during development. Therefore, we decided for 24 h substance exposure. Nevertheless, further studies are needed on in vivo pharmacokinetics of caffeine in childhood and adolescence. Interestingly, effects of single taurine treatment on immature oligodendrocyte degeneration were less pronounced compared to caffeine single and combined treatment. However, proliferation of A2B5-positive oligodendrocytes was significantly reduced, resulting in an overall decreased cell number. These results indicate that detrimental effects of taurine on developing oligodendrocytes might be rather attributed to inhibition of proliferation than induction of degeneration. This seems to be in contrast to previous reports where taurine was described as a putative neuromodulator, improving neurological dysfunctions, especially cognitive function [35,36]. On the other hand, in human lung cancer cells taurine treatment with different concentrations for 24 h, 48 h, and 72 h inhibited cell proliferation [37], which is in accordance to our observations in the present study. Further research will be needed to delineate positive and negative effects of taurine in cell- and disease-specific experimental settings.

The combined treatment of caffeine and taurine induced the most pronounced increase of oligodendrocyte degeneration compared to single treatments, which may indicate interactions between both compounds. Additive effects of caffeine and taurine have already been described in a study analysing cardiac parameters in athletes demonstrating exacerbated effects of single compounds after simultaneous uptake [38]. Furthermore, mice treated with caffeine and taurine for 2 weeks demonstrated a significantly enhanced endurance performance after a combined treatment compared to single treatments [39]. Even though an increased calcium-release in muscles or competitive inhibition of adenosine receptors expressed by different neuronal cells in the CNS has been discussed [39], the detailed underlying mechanisms of a potential interaction remain elusive and require further investigation.

Brain development is strongly dependent on oligodendrocyte maturation into myelinating cells. Therefore, we analysed differentiation capacity of immature oligodendrocytes demonstrating a significantly decreased number of mature MBP-positive oligodendrocytes three days after treatment. These results suggest that energy compounds not only induce acute cellular degeneration but that biological programs of surviving oligodendrocytes are severely disturbed. This is of particular importance, as myelination is the key for neuronal network formation [40,41]. Even though neurogenesis in most cortical and subcortical regions is supposed to be completed by postnatal-day 15 in rats and by 2.5 years in humans [9], neurogenesis lasts into adulthood in other brain regions, i.e., the hippocampus and olfactory bulbs [42,43]. Furthermore, during puberty neuronal network formation and integration is reorganized requiring competent oligodendrocytes to myelinate axons and to enable formation of new synapses [41]. 

In addition to competent myelinating oligodendrocytes, neuronal reorganisation during puberty depends on neuronal integrity, e.g., axonal network formation and dendritic branching [44]. Therefore, we analysed the effects of caffeine and taurine on neurons isolated from the hippocampus, the main structure of learning and memory formation involving adult neurogenesis and synaptogenesis [18]. In addition to neuronal cell loss immediately after treatment with caffeine and taurine, a significant reduction in number and length of total dendrites mainly caused by a reduction of higher dendrite branching was observed. These effects persisted for several days, which is of particular importance considering that dendritic arborisation coordinates with synaptic activity and is important for neural network integrity [45]. Furthermore, disturbed maintenance and disruption of dendrites and synapses has been associated with psychiatric illnesses, such as schizophrenia and major depressive disorders, as well as neurodegenerative diseases, such as Alzheimer’s disease [46]. Nevertheless, similarly as for oligodendrocytes, these results appear contradicting to previous findings, describing enhanced dendrite outgrowth in primary hippocampal neurons after taurine and caffeine treatment [47,48]. However, again concentrations applied, seem to be decisive. For example, protective effects of taurine on dendrite outgrowth were observed at 100 µM, while 2.7 mM, approximately equivalent to 0.34 mg/mL, was described to decrease neurite length and number [47,49] supporting our observations. Besides dendritic branching, axonal integrity is important for functionality and activity of neurons [50]. Analysis of axonal morphology demonstrated an increased number of axonal breaks and pearl-like structures three days after treatment with caffeine and taurine and especially in the combined treatment. Similar structures were also described in human neuronal SH-SY5Y cells incubated with caffeine and in an experimental model of Alzheimer’s disease [12,24]. This abnormal morphology was suggested to be caused by accumulation of proteins, organelles, and vesicles and to result in axonal defects and impaired axonal transport. In view of these previous studies, our observations suggest that high concentrations of caffeine and taurine in energy drinks do not only inhibit the capacity of dendritic branching but also disturb axonal function.

We have chosen high concentrations of caffeine and taurine corresponding to that of commercially available energy drinks. We are aware of the fact that concentrations applied in the present and previous [12] in vitro studies are higher than concentrations determined in plasma of humans and rodents after oral consumption/administration [32,33,51]. However, translation of in vitro doses to humans is always limited considering the complex variables, one cannot control for in vitro, e.g., co-consumption of other drugs like alcohol and smoking or of sugar provided in energy drinks which may affect pharmacokinetics. Our rationale was mainly based on the fact that consumption, especially in adolescents and young adults, is still rising. Twelve percent of adolescents and 13.4% of young adults have been identified as high acute consumers, with at least 1 L of energy drink consumption per session, and high chronic consumers, with 13.3% of young adults consuming an average volume of 4.5 L/month, as well as 12% of adolescents being high chronic consumers, with 4–5 sessions/week and an average volume of 7 L/month [22].

In the present study we provide evidence that an increased consumption of energy drinks may negatively influence neurodevelopment during childhood and adolescence, reducing immature oligodendrocyte survival and their differentiation capacity, which is accompanied by direct effects on neuronal integrity. Effects were analyzed in purified primary cell cultures. Even though pharmacokinetics of single energy drink compounds after oral intake can hardly be modeled in vitro, the rapid absorption of caffeine and taurine and unproblematic passing of the blood–brain barrier [52,53], indicate that high concentrations of caffeine and taurine reach the brain. In view of the continuous rise of teens and children consuming energy drinks further studies are needed to confirm these effects in vivo considering the complex interactions between different CNS cell types needed for oligodendrocyte development, myelination and axonal network formation during puberty.

## Supplementary Materials

The following are available online at https://www.mdpi.com/2073-4409/8/11/1381/s1, Figure S1: Caffeine and taurine reduce the number of hippocampal neurons. Table S1: Detailed results of statistical analyses for effects on oligodendrocytes. Table S2: Detailed results of statistical analyses for effects on hippocampal neurons.
