# Supplementary material for: Detrimental Impact of Energy Drink Compounds on Developing Oligodendrocytes and Neurons
Source: Cells. 2019 Nov 3;8(11):1381. doi: 10.3390/cells8111381 (PMC6912672; doi:10.3390/cells8111381)
Supplement: Supplementary file 1 [file cells-08-01381-s001.pdf]

**Immature oligodendrocyte**		**treatment**				***F*-statistics**	**p-value derived from one way ANOVA**	**p-value derived from Bonferroni post hoc test**				
Analysis, unit	Figure	Control (ct) Mean ± SD	Caffeine (caf) Mean ± SD	Taurine (ta) Mean ± SD	caffeine + taurine (caf + ta) Mean ± SD			ct vs. caf	ct vs. ta	ct vs. caf + ta	caf vs. caf + ta	ta vs. cat + ta
Olig2 cells/mm²	1B	314.2 ±40.12	192.2 ±60.26	188.6 ±59.62	188.1 ±68.08	$F_{3, 56}$ = 17.33	<0.0001	<0.0001	<0.0001	<0.0001	>0.9999	>0.9999
A2B5 area/mm²	1C	0.210814 ±0.039544	0.137515 ±0.046613	0.105316 ±0.039983	0.111146 ±0.048131	$F_{3, 56}$ = 18.43	<0.0001	0.0001	<0.0001	<0.0001	0.5215	>0.9999
PCNA⁺ cells of Olig2 [%]	2B	40.15 ±10.8	8.674±7.024	19.71 ±8.266	15.97 ±6.151	$F_{3, 56}$ = 40.10	<0.0001	<0.0001	<0.0001	<0.0001	0.0932	>0.9999
ratio cCaspase-3/ GAPDH	2C	1.00 ±0.1721	1.874 ±0.2655	1.208 ±0.4440	3.054 ±1.301	$F_{3, 12}$ = 6.886	0.0060	0.5253	>0.9999	0.0071	0.1780	0.0151
**Differentiated Oligodendrocytes**												
Olig2 cells/mm²	3B	368.9 ±61.30	300.3 ±56.29	278.1 ±40.82	248.3 ±60.34	$F_{3, 56}$ = 12.91	<0.0001	0.0063	0.0002	<0.0001	0.0635	0.7314
% MBP cells	3C	93.29 ±2.708	85.29 ±4.673	85.98 ±3.219	84.69 ±4.034	$F_{3, 56}$ = 17.43	<0.0001	<0.0001	<0.0001	<0.0001	>0.9999	>0.9999

Supplementary table 1: Detailed results of statistical analyses for effects on oligodendrocytes.

**Neurons 4d**		**treatment**						**p-value derived from Bonferroni post hoc test**					
**Analysis, unit**	**Figure**	**Control (ct) Mean ± SD**	**Caffeine (caf) Mean ± SD**	**Taurine (ta) Mean ± SD**	**caffeine + taurine (caf + ta) Mean ± SD**	***F*-statistics**	**p-value derived from one way ANOVA**	**ct vs. caf**	**ct vs. ta**	**ct vs. caf + ta**	**caf vs. caf + ta**	**ta vs. cat + ta**
Number total dendrites/cell	4B	5.758 ±2.865	3.944 ±1.818	3.313 ±1.361	3.825 ±1.855	$F_{3, 370}$ = 25.07	<0.0001	<0.0001	<0.0001	<0.0001	>0.9999	0.4784
Number primary dendrites/cell	4C	3.141 ±1.161	2.899 ±1.001	2.759 ±0.9317	2.864 ±1.048	$F_{3, 370}$ = 2.251	0.0820	0.5632	0.0716	0.2996	>0.9999	>0.9999
Number higher dendrites/cell	4D	3.866 ±2.322	2.514 ±0.9316	2.087 ±0.5146	2.622 ±1.320	$F_{3, 160}$ = 9.699	<0.0001	0.0007	0.0002	0.0016	>0.9999	>0.9999
Total dendrite length/cell [µm]	4E	240.6 ±98.65	170.0 ±83.77	154.3 ±76.92	187.4 ±82.16	$F_{3, 370}$ = 17.76	<0.0001	<0.0001	<0.0001	<0.0001	0.8219	0.0483
Primary dendrite length/cell [µm]	4F	140.7 ±75.17	132.9 ±65.83	133.1 ±70.10	154.5 ±75.70	$F_{3, 370}$ = 1.921	0.1258	>0.9999	>0.9999	0.8654	0.1922	0.2231
Higher dendrite length/cell [µm]	4G	147.6 ±87.25	89.34 ±41.79	80.03 ±38.16	89.00 ±56.11	$F_{3, 160}$ = 10.97	<0.0001	<0.0002	0.0003	0.0001	>0.9999	>0.9999
Map2 cells/mm²	Supple-mentary	131.6 ±92.21	79.21 ±55.96	80.45 ±73.89	58.86 ±32.59	$F_{3, 102}$ = 5.451	0.0016	0.0337	0.0303	0.0011	>0.9999	>0.9999

**Neurons d8**												
Number total dendrites/cell	5B	9.560 ±4.595	5.293 ±2.669	5.603 ±2.645	5.062 ±2.315	$F_{3, 312}$ = 34.89	<0.0001	<0.0001	<0.0001	<0.0001	>0.9999	>0.9999
Number primary dendrites/cell	5C	3.547 ±1.482	3.207 ±1.141	3.167 ±0.9989	3.123 ±1.100	$F_{3, 312}$ = 2.020	0.1111	0.3761	0.2458	0.1358	>0.9999	>0.9999
Number higher dendrites/cell	5D	6.731 ±3.800	3.353 ±2.217	3.725 ±2.011	3.413 ±1.939	$F_{3, 211}$ = 21.75	<0.0001	<0.0001	<0.0001	<0.0001	>0.9999	>0.9999
Total dendrite length/cell [µm]	5E	401.9 ±161.5	288.8 ±198.5	269.9 ±115.0	257.1 ±92.21	$F_{3, 312}$ = 15.35	<0.0001	<0.0001	<0.0001	<0.0001	>0.9999	>0.9999
Primary dendrite length/cell [µm]	5F	152.0 ±85.47	184.7 ±111.7	164.7 ±78.91	180.6 ±89.43	$F_{3, 312}$ = 2.061	0.1053	0.1390	>0.9999	0.2730	>0.9999	>0.9999
Higher dendrite length/cell [µm]	5G	279.8 ±165.8	167.5 ±148.2	160.8 ±94.24	134.7 ±76.62	$F_{3, 211}$ = 14.50	<0.0001	<0.0001	<0.0001	<0.0001	>0.9999	>0.9999

Supplementary table 2: Detailed results of statistical analyses for effects on hippocampal neurons.

Supplementary Figure S1

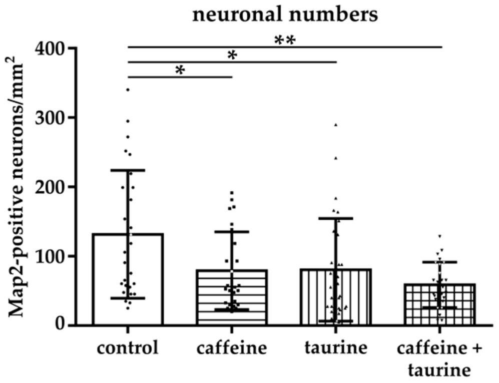

Supplementary Figure S1: Caffeine and taurine reduce the number of hippocampal neurons. After a culture-period of three days hippocampal neurons were treated with 0.3 mg/ml caffeine and 4 mg/ml taurine alone or in combination for 24 h. The density of hippocampal neurons was quantified by counting Map2/DAPI-double-positive cells. Data are derived from 24-30 images of three independent experiments. *$p<0.05$, **$p<0.01$, one-way ANOVA followed by a Bonferroni post hoc test.
